# Discovery of Macrocyclic Peptide Inhibitors Targeting MYC Oncoprotein via mRNA Display

**DOI:** 10.3390/ph19060967

**Published:** 2026-06-22

**Authors:** Jinzhu Chen, Fanglin Li, Chenguang Yuan, Xiaoling Geng, Yu Zhang, Qiurong Ding, Yan Chen

**Affiliations:** 1 Shanghai Institute of Nutrition and Health, University of Chinese Academy of Sciences, Chinese Academy of Sciences, Shanghai 200031, China; chenjinzhu2022@sinh.ac.cn (J.C.);; 2 Shenzhen Sungening Biological Co., Ltd., Shenzhen 518172, China; 3 School of Life Science, Hangzhou Institute for Advanced Study, University of Chinese Academy of Sciences, Hangzhou 310024, China

**Keywords:** mRNA display, MYC, macrocyclic peptide, tumor treatment, undruggable target

## Abstract

**Background/Objectives:** mRNA display technology has emerged as a powerful platform for discovering macrocyclic peptides against intractable proteins. However, direct screening against the “undruggable” transcription factor MYC using this approach remains largely unexplored. In this study, we aimed to integrate tyrosinase-mediated cyclization with mRNA display to identify novel macrocyclic peptide inhibitors targeting MYC. **Methods:** We performed mRNA display combined with tyrosinase-mediated cyclization to generate macrocyclic peptides targeting MYC. Antiproliferative activity was assessed in MYC-dependent tumor cells using CCK8 assay. C-terminal fusions with a TAT-derived cell-penetrating peptide were generated to enhance cell membrane permeability. Binding affinities were measured by bio-layer interferometry (BLI). MYC transcriptional activity was evaluated by RNA sequencing (RNA-seq) analysis of canonical MYC target genes. **Results:** The identified macrocyclic peptides exhibited potent antiproliferative activity against MYC-dependent tumor cells, with half-maximal inhibitory concentration (IC_50_) values in the micromolar range. Fusion with the TAT peptide improved antiproliferative potency, yielding IC_50_ values of 1–3 μM in MYC-dependent cell lines. BLI assays confirmed dose-dependent binding of the peptides to MYC, with dissociation constants (Kd) in the micromolar range. Furthermore, RNA-seq analysis revealed significant downregulation of canonical MYC target genes upon treatment with the TAT-fusion macrocyclic peptide, indicating specific suppression of MYC transcriptional activity. **Conclusions:** This work establishes the feasibility of using mRNA display to target the “undruggable” protein MYC and identifies a panel of macrocyclic peptides as promising lead candidates for further optimization toward targeted therapies for MYC-driven cancers.

## 1. Introduction

MYC is one of the most frequently dysregulated oncogenes in human cancers [1,2]. However, its intrinsic disorder and lack of well-defined binding pockets have long made it “undruggable” [2,3,4,5]. Multiple therapeutic strategies targeting MYC have been explored, but each has inherent limitations [4,6,7,8]. For instance, small-molecule inhibitors like 10058-F4 and 10074-G5 [9,10], identified through high-throughput screening, suffer from low affinity, poor cellular permeability, and limited selectivity. Similarly, the miniprotein Omomyc (OMO-103) [11] has its own drawbacks including limited cell-penetrating ability, the need for high concentrations to exhibit activity, and susceptibility to proteolytic degradation. In addition, PROTAC-based degraders such as WBC100 face challenges in aspects like delivery efficiency, potential resistance, and complex pharmacokinetics [12,13]. These limitations highlight the urgent need for novel therapeutic approaches integrating high binding affinity, proteolytic stability, and efficient cellular uptake.

The technology of mRNA display has become a powerful platform for finding macrocyclic peptide ligands for challenging protein targets [14,15,16,17,18,19]. With its advantages of large library capacity (10^12^–10^15^), complex cyclic peptide libraries, and fully in vitro operation, mRNA display technology offers a powerful and straightforward tool for targeting “undruggable” proteins which lack traditional small-molecule binding pockets [13,20,21,22]. Despite these technical advances, the direct application of mRNA display to screen for macrocyclic peptides specifically targeting MYC has remained unexplored [21,23,24,25,26].

Here, we report the integration of tyrosinase-mediated cyclization with mRNA display to identify macrocyclic peptide ligands targeting MYC [17]. The selected peptides exhibited micromolar antiproliferative activity against MYC-dependent tumor cells. To overcome the membrane permeability limitations inherent to cyclic peptides, we generated C-terminal fusions with a TAT-derived cell-penetrating peptide [27]. This modification greatly enhanced antiproliferative potency. Direct binding to MYC was confirmed by bio-layer interferometry (BLI). Furthermore, transcriptomic analysis revealed that treatment with the lead peptide TAT-CP5 led to significant downregulation of canonical MYC target genes, confirming specific inhibition of MYC transcriptional activity. Taken together, this work establishes mRNA display as a viable strategy for targeting MYC and identifies a series of macrocyclic peptides as promising lead candidates for cancer therapy in MYC-overexpressing cells.

## 2. Results

### 2.1. mRNA Display Selection of Macrocyclic Peptides Targeting MYC

We first expressed and purified recombinant full-length human MYC protein in *E. coli* for mRNA display selections. The MYC coding sequence was cloned into a pET28a vector with an N-terminal 6xHis tag for nickel affinity purification and a C-terminal Avi tag for enzymatic biotinylation (Figure S1). We combined tyrosinase-catalyzed macrocyclization with mRNA display to select for macrocyclic peptides that bind to MYC [17,28,29].

**Figure 1:**
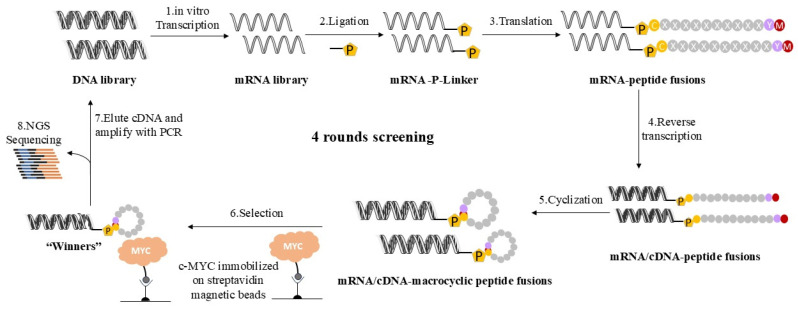
Schematic overview of the mRNA display selection for MYC-binding macrocyclic peptides. The different colors in sequencing image indicate fixed and random DNA sequences.

Our selection began with a DNA library encoding peptides with an N-terminal tyrosine, ten randomized amino acids (“NNK” codons) in between, and a C-terminal cysteine, enabling head-to-tail cyclization via tyrosinase (megTYR) catalysis [17]. The library capacity was approximately 10^13^, providing sufficient diversity for selecting high-affinity MYC-binding ligands. Following in vitro transcription, the mRNA was ligated to a Puromycin-linker (P-linker) and translated using the PURExpress system to generate mRNA-peptide fusions. After reverse transcription, the library was then treated with megTYR to rapidly be cyclized to create mRNA/cDNA–macrocyclic peptide fusions. The macrocyclic peptides library was subjected to four rounds of affinity selection against biotinylated MYC immobilized on streptavidin-coated magnetic beads. Bound mRNA/cDNA–macrocyclic peptides were eluted, amplified by PCR, and carried forward to the next selection round. After the final round of selection, the enriched sequences were determined by next-generation sequencing (Figure 1 and Figure S2).

A DNA library was transcribed, ligated to a puromycin linker (P-linker), and translated to generate mRNA–peptide fusions. After reverse transcription, Tyrosinase-catalyzed peptide macrocyclization between N-terminal tyrosine and C-terminal cysteine. The library underwent four rounds of selection on streptavidin-immobilized MYC. Enriched sequences were amplified by PCR and identified by NGS.

### 2.2. Identification of Enriched Macrocyclic Peptide Sequences by NGS

Following four rounds of selection against MYC, the enriched DNA libraries from each round (Round 0–4) were subjected to next-generation sequencing (NGS) using the Illumina MiSeq platform (2 × 150 bp) [30]. Raw FASTQ files were processed using a custom Python script (Python 3.13.6) to extract sequences corresponding to the peptide coding regions, with quality filtering to retain only reads containing perfect matches to the flanking constant regions upstream of the start codon and downstream of the random region [31].

**Figure 2:**
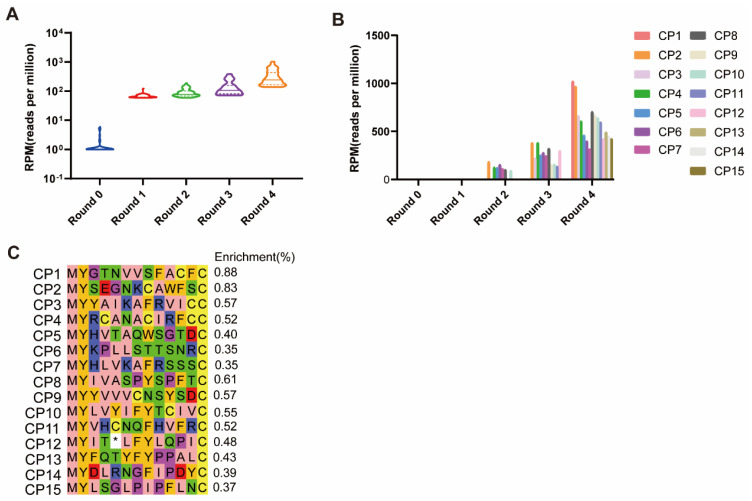
Sequence analysis of enriched macrocyclic peptides from mRNA display selection against MYC. (**A**) Violin plots showing the reads per million (RPM) for the top 50 sequences across selection rounds 0–4. (**B**) The RPM values of the top 15 macrocyclic peptide sequences across selection rounds 0–4. (**C**) List of the top 15 enriched macrocyclic peptide sequences with their corresponding enrichment percentages. The different colors indicate different amino acids. * indicates a stop codon.

Sequence abundance was quantified as reads per million (RPM) for each unique sequence. To evaluate the dynamic changes in enrichment throughout the selection process, we analyzed the top 50 ranked sequences from each round. As shown in Figure 2A, violin plots of the RPM distributions revealed a progressive increase in enrichment from Round 0 to Round 4, with notable enrichment beginning at Round 1 and reaching maximum levels at Rounds 4. To identify candidate sequences for further validation, we analyzed the RPM values of the top 15 macrocyclic peptides across selection rounds. Those showing progressive enrichment were selected and visualized by histogram (Figure 2B). A clear round-by-round increase in enrichment was observed for these sequences, suggesting their potential binding affinity to MYC. Furthermore, Figure 2C presents the top 15 most abundant sequences and their enrichment. Among them, cyclic peptide 1 (CP1) (MYGTNVVSFAFCFC) had the highest enrichment, with a percentage of 0.88, followed by CP2 (MYSENKCAWFSCC; 0.83%) and so on. The top 15 sequences showed a clear enrichment, ranging from 0.35% to 0.88% (Figure 2C).

Together, the progressive enrichment of these sequences across selection rounds suggests selective binding affinity for MYC. Based on their enrichment profiles and sequence diversity, we selected and synthesized seven sequences of interest as lead candidates. Following tyrosinase-mediated macrocyclization, these macrocyclic peptides were prepared for further validation studies (Table S2, Figure S3). Notably, CP3 was excluded from subsequent studies due to its poor cyclization efficiency.

### 2.3. Functional Validation of Selected Macrocyclic Peptides in MYC-Dependent Cancer Cells

**Figure 3:**
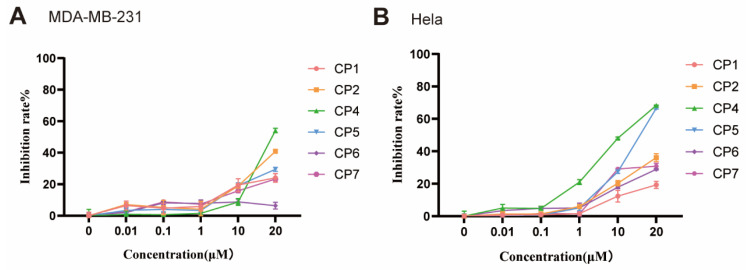
Macrocyclic peptides identified through mRNA display exhibit selective antiproliferative activity against MYC-dependent cancer cells. (**A**) MDA-MB-231 and (**B**) HeLa cells were treated with increasing concentrations of macrocyclic peptides CP1–CP7 without CP3 (0, 0.01, 0.1, 1, 10, and 20 μM) for 72 h, and inhibition rate was assessed using the CCK-8 assay. Experimental conditions were the same as described above. All data are presented as mean ± SEM (*n* = 3).

To evaluate the biological activity of the enriched macrocyclic peptides, we assessed their antiproliferative effects on MYC-dependent cancer cells using Cell Counting Kit-8 assay (CCK-8 assay). As showed in the Figure 3A,B, six macrocyclic peptides (CP1–CP7, without CP3) were tested for antiproliferative inhibition rate in MYC-dependent MDA-MB-231 and HeLa cells, which were exposed to different macrocyclic peptide concentrations (0.01–20 μM) for 72 h. We observed that all macrocyclic peptides inhibited the proliferation of both cell lines in a concentration-dependent manner. At low concentrations (0.01–1 μM), the macrocyclic peptides showed almost no activity. However, at higher concentrations (1–20 μM), their antiproliferative effects varied considerably. Moreover, HeLa cells were generally more sensitive than MDA-MB-231 cells, consistent with the differential MYC dependence of these cancer cell lines. Notably, CP2, CP4, and CP5 exhibited significant antiproliferative activity at concentrations above 10 μM, with potency comparable to or exceeding that of previously reported anti-MYC inhibitors such as GT19630 which is a molecular glue degrading both MYC and GSPT1, OMO-103 which is a MYC inhibitor consisting of a 91-amino acid miniprotein and a synthetic transcriptional repressor derived from the bHLH domain of MAX [8,11,32,33].

Collectively, these results demonstrate that the macrocyclic peptides identified through mRNA display exhibit selective antiproliferative activity against MYC-dependent cancer cells, with CP2, CP4 and CP5 emerging as the most promising candidates for further development. The differential activity profiles between MDA-MB-231 and HeLa cells may reflect cell line-specific variations in MYC signaling pathways or peptide uptake efficiency.

### 2.4. Determination of Binding Affinities of Macrocyclic Peptides for MYC

**Figure 4:**
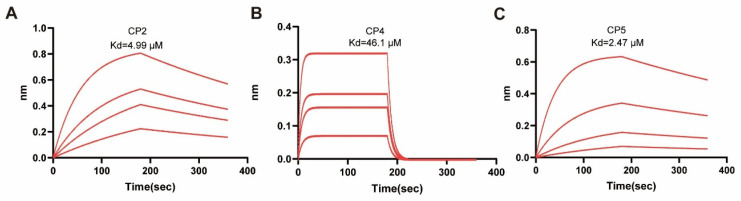
Binding affinity of macrocyclic peptides to MYC. The binding of (**A**) CP2, (**B**) CP4 and (**C**) CP5 to immobilized MYC protein was measured in a concentration-dependent manner.

To quantitatively characterize the binding affinity of the candidate macrocyclic peptides CP2, CP4, and CP5 to MYC, we performed real-time interaction analysis using Bio-Layer Interferometry (BLI) [34]. Purified MYC was biotinylated and immobilized onto streptavidin (SA) biosensors. The sensors were then exposed to various concentrations of each macrocyclic peptides (CP2, CP4, and CP5) to measure association, followed by a dissociation step in buffer. As shown in Figure 4, the macrocyclic peptides exhibited concentration-dependent binding to MYC: CP2 and CP5 displayed the highest affinity with Kd values of 4.99 μM and 2.47 μM, Compared to CP2 and CP5, CP4 exhibited a faster association and dissociation, suggesting a weaker binding affinity (Kd = 46.1 μM). Additionally, we performed the same BLI assay using bovine serum albumin (BSA) as an irrelevant control protein. As shown in Figure S4, under identical experimental conditions, no detectable binding to BSA was observed for CP2, CP4, or CP5. These results demonstrate that the interaction between the macrocyclic peptides and MYC is specific and not due to non-specific protein-binding artifacts, supporting the selectivity of these peptides for MYC over an unrelated control protein.

### 2.5. Cell-Penetrating Peptide Mediated Intracellular Delivery of Macrocyclic Peptides

To enhance cellular uptake of the selected macrocyclic peptides and facilitate their engagement with the intracellular target MYC, we adopted a conjugation strategy utilizing a well-characterized cell-penetrating peptide (CPP) [35,36,37]. The TAT peptide (GRKKRRQRRR), derived from the transactivator of transcription protein of human immunodeficiency virus, is one of the most widely studied CPPs and has been shown to efficiently deliver a variety of cargoes into mammalian cells [35]. We hypothesized that fusion of this cationic, arginine-rich sequence to our macrocyclic peptides would promote cellular internalization while preserving the target-binding activity of the peptide moiety [38]. Therefore, we engineered CPP conjugates by fusing the HIV-1 TAT-derived sequence GRKKRRQRRR to the C-terminus of lead candidates CP2 and CP5 (designated TAT-CP2 and TAT-CP5, respectively). The antiproliferative inhibition rate of these conjugates were evaluated on cancer cells using CCK-8 assays, with concentration ranging from 0.01 to 20 μM.

**Figure 5:**
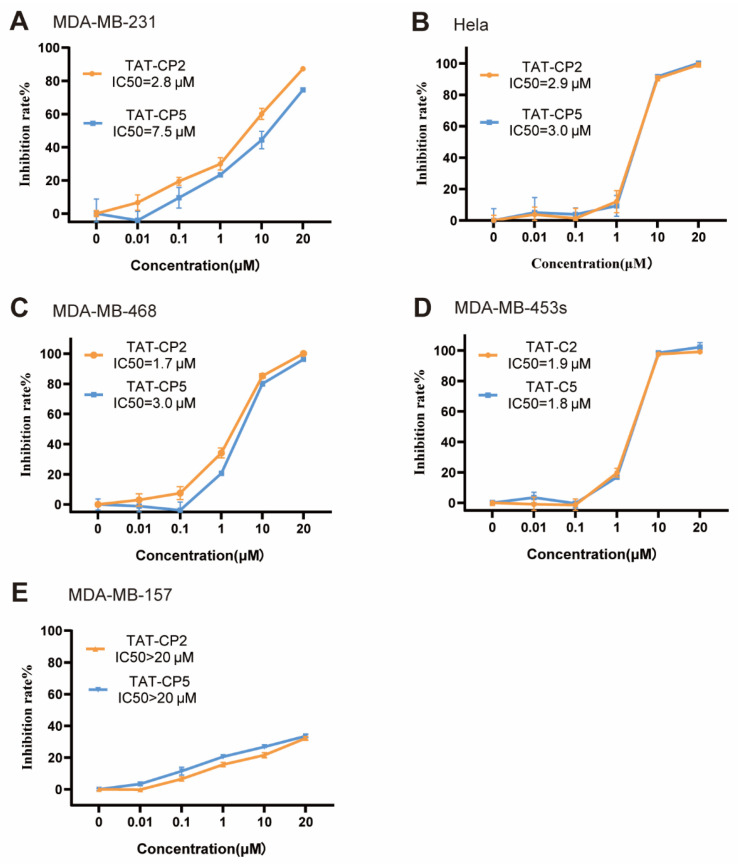
CPP conjugation enhances the antiproliferative inhibition rate of macrocyclic peptides. (**A**) MDA-MB-231, (**B**) HeLa, (**C**) MDA-MB- 468, (**D**) MDA-MB-453s cells were treated with increasing concentrations of macrocyclic peptides TAT-CP2 and TAT-CP5 (0, 0.01, 0.1, 1, 10, 20 μM) for 72 h, and inhibition rate was assessed using the CCK-8 assay. (**E**) Inhibitory effects of TAT-CP2 and TAT-CP5 on proliferation of MYC-low (MDA-MB-157) cells. Experimental conditions were the same as described above. All data are presented as mean ± SEM (*n* = 3).

As shown in Figure 5A–D, we observed that both TAT-conjugated peptides exhibited dose-dependent antiproliferative inhibition rate across all cell lines, achieving >50% growth inhibition rate at 10 μM, with the majority of IC50 at 1–3 mM for TAT-CP2 and TAT-CP5. Compared to the results in Figure 3, the unconjugated macrocyclic peptides CP2 and CP5 showed only ~20% inhibition at 10 mM, while TAT-CP2 and TAT-CP5 had 80–100% inhibition at this concentration. Furthermore, to determine whether the growth inhibitory effects of macrocyclic peptides depend on MYC expression levels, we compared TAT-CP2 and TAT-CP5 activity in MDA-MB-157 (MYC-low) breast cancer cells. MDA-MB-157 was selected as a MYC-low model based on a previous study showing that this cell line is MYC-independent [33]. As shown in Figure 5E, treatment with TAT-CP2 and TAT-CP5 resulted in significantly weaker inhibition in MYC-low cells than in MYC-high cells. This differential sensitivity suggests that high MYC expression sensitizes breast cancer cells to TAT-CP2 and TAT-CP5 induced growth suppression, supporting a MYC-dependent mechanism of action.

These results demonstrate that TAT fusion substantially enhances the antiproliferative potency of the selected macrocyclic peptides, improving their therapeutic efficacy by more than 3-fold to enable effective intracellular targeting of MYC.

### 2.6. Macrocyclization Confers Antiproliferative Activity to TAT-Conjugated Peptides

**Figure 6:**
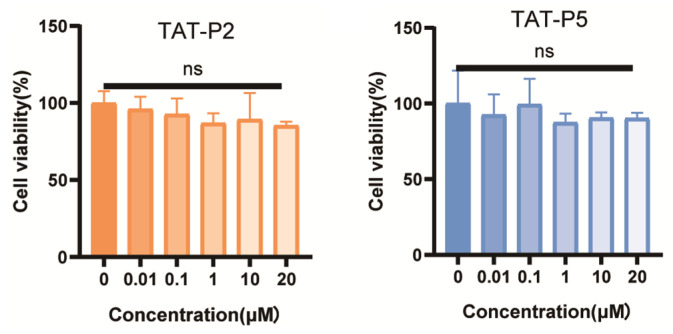
Macrocyclization confers antiproliferative activity to TAT-conjugated peptides (ns: non-significant).

Given that TAT conjugation facilitated cellular delivery and enhanced antiproliferative activity, we next asked whether macrocyclization itself is required for this effect. To address this question, we used the corresponding linear peptides (uncyclized, designated TAT-P2 and TAT-P5) for direct comparison with their cyclic counterparts. As shown in Figure 6, TAT-conjugated linear peptides failed to elicit significant antiproliferative effects across all concentrations (0.01–20 μM) in MYC-dependent cancer cells. Cell viability remained 80–90% even at the highest concentration (20 μM), with no dose-dependent reduction observed. In contrast, the corresponding TAT-conjugated macrocyclic peptides exhibited potent growth inhibition at 10 μM (Figure 5). These results demonstrate that the macrocyclic conformation is critical for biological activity, and that linearization completely abrogates the antiproliferative function despite the presence of the same amino acid sequence and TAT-mediated cellular uptake. This result demonstrates that cyclization is critical for maintaining the bioactive conformation necessary for target binding and function.

Cell viability of Hela cell treated with increasing concentrations (0, 0.01, 0.1, 1, 10, 20 μM) for 72 h of TAT-conjugated linear peptides (TAT-P2 and TAT-P5). Data are presented as mean ± SEM (*n* = 3).

### 2.7. TAT-CP5 Specifically Suppresses MYC Activity in MYC-Overexpressing Cells

**Figure 7:**
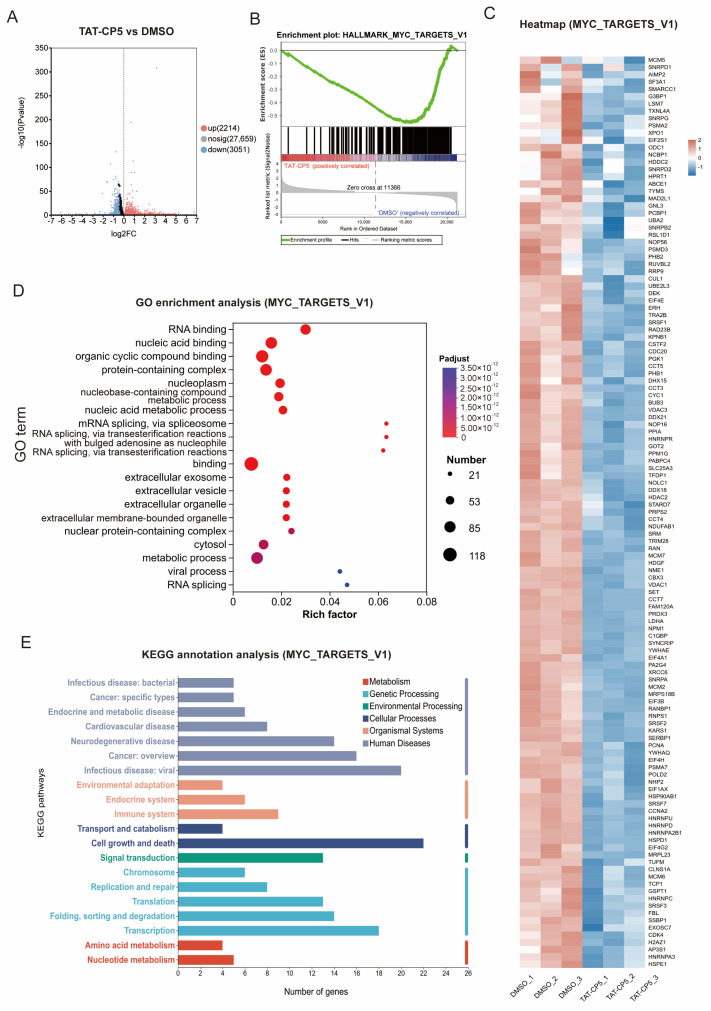
TAT-CP5 specifically suppresses MYC transcriptional activity. (**A**) Volcano plot showing differentially expressed genes in MYC-overexpressing HEK293T cells. Genes significantly downregulated and upregulated are shown in light blue and light red, respectively; genes from the HALLMARKMYC TARGETS V1gene set are colored dark black. (**B**) Gene set enrichment analysis (GSEA) result showing that the HALLMARKMYC TARGETS V1gene signatures were significantly enriched in DMSO-treated cells. NES, normalized enrichment score: −2.708; *p*-value: <0.001; FDR, false discovery rate: <0.001. (**C**) Heatmap displaying expression levels of HALLMARK MYC Target genes between DMSO control and TAT-CP5-treated group. (**D**) Gene Ontology (GO) enrichment analysis of MYC target genes. (**E**) KEGG pathway analysis of MYC target genes.

To test whether TAT-CP5 directly affected MYC activity, we performed RNA-seq on the MYC-overexpressing HEK293T cells and evaluated the differentially expressed genes using DESeq2. As shown in Figure 7A, TAT-CP5 treatment caused significant changes in the transcriptome, with 2214 genes significantly downregulated and 3051 genes upregulated, consistent with MYC’s master transcription factor role. Notably, most MYC target genes were consistently downregulated (negative log_2_FC) upon TAT-CP5 treatment, indicating that TAT-CP5 suppresses MYC transcriptional activity. Gene set enrichment analysis (GSEA) showed that the HALLMARK MYC Targets V1 gene signature [39] was significantly enriched in DMSO treated cells, as indicated by a strong negative enrichment score and low false discovery rate (NES = −2.708, *p* < 0.001, FDR < 0.001). This finding demonstrates that MYC target genes are preferentially downregulated following TAT-CP5 treatment, providing further evidence that MYC pathway is suppressed by TAT-CP5 treatment (Figure 7B). Consistent with the GSEA results, the heatmap result revealed a marked downregulation of canonical MYC target genes across all biological replicates following TAT-CP5 treatment. These data collectively demonstrate that TAT-CP5 functionally inhibits MYC transcriptional activity (Figure 7C). GO analysis demonstrated significant enrichment of MYC-regulated processes, including RNA binding, nucleic acid metabolism, mRNA splicing, and ribonucleoprotein complex biogenesis (Figure 7D), consistent with MYC’s role in promoting proliferation. KEGG analysis further showed that downregulated genes were enriched in cell growth and death pathways (Figure 7E), many of which represent canonical MYC targets. The suppression of these pathways provides a mechanistic basis for the antiproliferative effects of TAT-CP5, confirming that it inhibits cancer cell proliferation by disrupting MYC-driven transcriptional programs.

## 3. Discussion

Unlike well-folded proteins, MYC is intrinsically disordered and lacks stable binding pockets, which severely limits structure-based computational methods such as molecular docking [1,4]. These approaches rely on predefined three-dimensional structures and cannot adequately capture the conformational dynamics of MYC. In contrast, mRNA display enables unbiased, affinity-driven selection from ultra-large macrocyclic peptide libraries without any prior structural knowledge. This experimental strategy is particularly suited for challenging targets like MYC. Therefore, the present study provides a practical demonstration that mRNA display can overcome the inherent limitations of in silico approaches for intrinsically disordered transcription factors.

In this study, we successfully employed mRNA display to discover a series of macrocyclic peptides targeting the “undruggable” transcription factor MYC. The lead peptides exhibited micromolar antiproliferative activity against MYC-dependent cancer cells, which was substantially enhanced by TAT-mediated intracellular delivery. Transcriptomic profiling confirmed that TAT-CP5 functionally inhibits MYC transcriptional activity, leading to downregulation of canonical MYC target genes and suppression of MYC-driven oncogenic pathways.

However, while our results are promising, several questions remain to be addressed. First, the precise binding site of these peptides on MYC remains unclear. MYC is intrinsically disordered, lacking a stable three-dimensional structure, which poses inherent challenges for conventional structure-based drug design targeting this oncoprotein [5]. Its bHLH-LZ domain, particularly the c-Myc (370–409) region, has been extensively studied as a druggable region [32]. MYC-MAX heterodimerization inhibitors such as MYCMI-6 and MYCi975, as well as the dominant-negative Omomyc peptide, are known to act by targeting this region and have been shown to interfere with MYC activity with various mechanisms [9,11,40,41]. Interestingly, our co-immunoprecipitation experiment (Figure S5) showed that the macrocyclic peptides did not visibly disrupt MYC–MAX binding, suggesting a mode of action distinct from these reported dimerization inhibitors. Second, the extent to which RNAseq-based transcriptomic changes reflect direct MYC inhibition versus secondary cellular responses requires further delineation. MYC is a master transcriptional regulator; its prolonged inhibition can trigger cascades of indirect gene expression changes, which may confound the interpretation of the RNAseq data [13]. The absence of chromatin immunoprecipitation (ChIP) data or single-cell resolution analyses prevents us from distinguishing direct target engagement from downstream consequences. Third, the mechanism by which TAT conjugation enhances antiproliferative activity of our cyclic peptides needs further investigation to prove that such effect is directly due to improvement of cellular uptake. Furthermore, future studies will be necessary to map the precise binding interface using domain mapping or structural approaches, and to assess direct DNA recruitment of MYC to definitively characterize the mechanism of action for these cyclic peptides. Nevertheless, our study establishes mRNA display as a viable strategy for the discovery of macrocyclic peptide inhibitors targeting MYC and provides promising lead candidates for further optimization toward MYC-driven cancer therapy.

## 4. Materials and Methods

### 4.1. Materials

The following reagents were obtained from commercial sources. KOD-Plus-Neo PCR kit and PCR purification kit were purchased from TOYOBO (Osaka, Japan) and Beyotime Biotechnology (Shanghai, China), respectively. Beyotime Biotechnology also supplied 25 mM NTP mix, RNase inhibitor, and DTT. New England Biolabs (NEB) (Ipswich, MA, USA) was the supplier for T7 RNA polymerase, Monarch RNA purification kit, 2× RNA loading dye, T4 RNA ligase 1, T4 PNK, and PURExpress ΔRF123 in vitro translation kit. Takara Bio (Kusatsu, Japan) provided 10 mM dNTPs. Urea, RNase A, and DNase I were obtained from Yeasen Biotechnology (Shanghai, China). SYBR Gold dye was sourced from Thermo Fisher Scientific (Waltham, MA, USA). Promega Corporation (Madison, WI, USA) supplied RQ1 RNase-Free DNase and M-MLV reverse transcriptase (RNase H minus). PD MiniTrap G-25 columns were purchased from Cytiva (Marlborough, MA, USA). Tropolone, Ni-NTA agarose, and streptavidin magnetic beads were acquired from MedChemExpress (MCE). All oligonucleotide primers were synthesized by Tsingke Biotechnology (Beijing, China). The pLVXm-N-FLAG-MYC plasmid used in this study was maintained in our laboratory.

### 4.2. Recombinant Protein Expression and Purification

The cDNA encoding MYC-AviTag was cloned into pET28a at BamHI/NotI sites. This plasmid encodes an N-terminal 6xHis on the protein of interest. 6xHis-Avi-tagged proteins were produced in the *E. coli* BL21 (DE3). Cultures were grown in Luria–Bertani (LB) at 37 °C to an OD_600_ of 0.6–0.8 before induction with 0.5 mM IPTG for 16 h at 37 °C. Cells were harvested and lysed with Lysis buffer (50 mM Tris, pH 7.5, 300 mM NaCl, 1 mM DTT, DNase, 1 mg/mL lysozyme and 1xPMSF). 6xHis-Avi-tagged proteins was purified at 4 °C using a BeyoGold™ His-tag Purification Resin (Beyotime Biotechnology, Shanghai, China) and eluted with 50 mM Tris, pH 7.5, 300 mM NaCl, 1 mM DTT, 5% glycerol, 1xPMSF and 250 mM imidazole. Following refolding by slow dialysis, the Avi-tagged protein was biotinylated in vitro with BirA for downstream mRNA Display selection. Recombinant megTYR protein with an N-terminal His-tag was expressed in Escherichia coli, purified by affinity chromatography, and was kindly provided by Professor Zhang Yi from the Laboratory of Nutritional Engineering at the Shanghai Institute of Nutrition and Health, Chinese Academy of Sciences.

### 4.3. Protocol of mRNA Display

Linear DNA library construction: Linear double-stranded DNA (dsDNA) libraries were generated by annealing two complementary single-stranded DNA (ssDNA) oligonucleotides (synthesized by Tsingke Biotechnology; see Table S1), followed by primer extension using KOD DNA polymerase. The resulting dsDNA products were purified with a PCR Cleanup Kit (Beyotime Biotechnology) and quantified using a NanoDrop 2000c spectrophotometer (Thermo Fisher Scientific).

In vitro transcription and P-Linker ligation: In vitro transcription was carried out with T7 RNA Polymerase (NEB) using approximately 1 μg of linear DNA library as template. The reaction was incubated at 37 °C for 6 h, after which 1 U of RQ1 RNase-Free DNase (Promega) was added to remove the DNA template according to the manufacturer’s instructions. The resulting RNA transcripts were purified with the Monarch RNA Cleanup Kit (NEB). To facilitate ligation with the P-Linker, the mRNA library was first annealed to the linker oligonucleotide. A 100 μL annealing reaction was assembled containing 4 μM transcribed mRNA, 8 μM P-Linker (Table S1), and T4 ligation buffer (NEB). The mixture was heated to 90 °C for 30 s and then slowly cooled to room temperature at a rate of 1 °C per second to ensure proper hybridization. Subsequently, ATP (1 mM final concentration), 10 U of T4 PNK, and 30 U of T4 RNA Ligase 1 (all from NEB) were added, and the ligation reaction was incubated at 25 °C for 30 min.

Gel electrophoresis and ethanol precipitation: Linear DNA libraries were analyzed by electrophoresis on a 2% agarose gel prepared in Tris-acetate-EDTA (TAE) buffer, run at a constant voltage of 250 V. For RNA analysis, transcribed mRNA libraries and mRNA-P-Linker ligation products were resolved on 8 M urea–6% polyacrylamide gels in Tris-borate-EDTA (TBE) buffer at 200 V. Gels were stained with SYBR Gold (Thermo Fisher Scientific) for visualization of RNA species. For isolation of the ligated product, gel slices containing the mRNA-P-Linker band were excised under blue light using a clean scalpel (GelBlew, CAVOY). RNA was recovered from the gel by crushing the slices and eluting overnight at 4 °C in 0.3 M NaCl with gentle agitation. The eluate was collected and subjected to ethanol precipitation. Specifically, sodium acetate (3 M, pH5.2) was added to a final concentration of 0.3 M, followed by three volumes of ice-cold 99.5% ethanol. The mixture was incubated at −80 °C overnight and then centrifuged at 12,000 rpm for 30 min at 4 °C. The supernatant was carefully removed, and the pellet was washed twice with 70% ethanol by centrifugation at 12,000 rpm for 10 min at 4 °C. After the final wash, the pellet was air-dried at room temperature for 5 min, resuspended in RNase-free water, and quantified using a NanoDrop 2000c spectrophotometer.

In vitro translation and reverse transcription: In vitro translation was performed in a 150 μL reaction volume using the PURExpress ΔRF123 system (NEB) following the manufacturer’s instructions. The mixture was incubated at 37 °C for 30 min, followed by 15 min at room temperature. EDTA (pH 8.0) was then added to a final concentration of 16.7 mM, and the reaction was incubated at 37 °C for an additional 30 min. After translation, the mRNA-P-Linker-peptide fusion products were reverse transcribed. The reaction mixture contained 250 μM dNTPs, 2 μM RT-PCR reverse primer (Table S1), 25 mM Tris-HCl (pH 8.3), 15 mM Mg(OAc)_2_, 10 mM KOH, and 1× M-MLV Reverse Transcriptase (RNase H Minus, Promega). Incubation was carried out at 42 °C for 1 h. Each sample was subsequently buffer-exchanged into tyrosinase reaction buffer (20 mM phosphate, pH 6.5) using a PD MiniTrap G-25 column (Cytiva) and diluted to a final volume of 300 μL with the same buffer. Recombinant megTYR was added to a concentration of 1 μM, and the mixture was incubated at room temperature for 1 h with rotation. The reaction was then diluted with 30 μL of 11× binding buffer (0.5 M Tris-HCl pH 7.5, 1.5 M NaCl, 110 mM MgCl_2_) and terminated by the addition of tropolone to a final concentration of 1.9 mM. For purification, 150 μL of Ni-NTA agarose resin (MCE) was added to the sample, and the suspension was rotated at 4 °C for 30 min. The resin was collected by spin column filtration, and the flow-through containing the unbound fraction was retained. Finally, this supernatant was buffer-exchanged into selection buffer (50 mM phosphate pH 7.2, 150 mM NaCl, 0.05% Tween 20, 1 mM DTT) and adjusted to a final volume of 500 μL.

Screening of macrocyclic peptides targeting MYC: For each round of selection, 100 μL of streptavidin-coated magnetic beads were washed twice with 1 mL of Wash Buffer and subsequently incubated with 200 μM biotinylated antibody or protein in 1 mL of Wash Buffer (bead concentration: 1 mg/mL) for 60 min at 4 °C with rotation to immobilize the target. Unbound material was removed by three washes with Wash Buffer prior to selection. The mRNA/cDNA–peptide fusions (500 μL in Selection Buffer) were then added to the target-immobilized beads and incubated overnight at 4 °C with rotation. Following incubation, the beads were washed three times with 1 mL of Wash Buffer. To recover the bound cDNA, the beads were resuspended in 100 μL of 1× PCR buffer and heated to 95 °C. The supernatant was immediately collected and subjected to amplification using the KOD-Plus-Neo PCR kit (TOYOBO) with the primers listed in Table S2. PCR cycles were performed until product bands were visible on a 2% agarose gel prepared in Tris-acetate-EDTA (TAE) buffer, typically requiring 15–35 cycles. For subsequent selection rounds, the library was pre-incubated with biotinylated beads alone prior to incubation with the immobilized MYC target, serving as a negative selection to deplete bead-binding sequences.

### 4.4. In Vitro Peptide Cyclization and MALDI-TOF

In vitro peptide cyclization was performed by incubating 100 μM peptides with 35 μM megTYR in 20 mM phosphate buffer (pH 6.5) at room temperature for 30 min. For MALDI-TOF MS analysis, cyclized samples were mixed 1:1 (*v*/*v*) with saturated α-cyano-4-hydroxycinnamic acid (CHCA) matrix solution (10 mg/mL in 50% acetonitrile/0.1% TFA) and spotted onto a MALDI target plate for analysis.

### 4.5. Cell Viability Assays

Cells were seeded in 96-well plates at 4000 cells per well and incubated overnight. Cells were then treated with various concentrations of macrocyclic peptides for 72 h. Cell viability was assessed using Cell Counting Kit-8 (CCK-8) (MeilunBio, Shanghai, China) according to the manufacturer’s protocol. Briefly, 100 µL of CCK-8 reagent was added to each well and incubated at 37 °C for 1 h. Absorbance was measured at 450 nm using a microplate reader. Each treatment was performed in triplicate.

### 4.6. Bio-Layer Interferometry (BLI)

Binding kinetics were measured by bio-layer interferometry (BLI) using a Gator instrument. Biotinylated cMYC protein was immobilized onto SAXT biosensors by dipping into 0.1 mg/mL protein solution for 60 s, followed by equilibration in PBST buffer (PBS + 0.05% Tween-20). Cyclic peptides at different concentrations were then associated for 120 s and dissociated in PBST for 180 s. The association and dissociation times will be adjusted according to the specific experimental conditions. All measurements were performed at 25 °C with shaking at 1000 rpm. Data were analyzed using a 1:1 binding model after double-reference subtraction.

## Data Availability

The original contributions presented in this study are included in the article/Supplementary Materials. Further inquiries can be directed to the corresponding author.

## Supplementary Materials

The following supporting information can be downloaded at: https://www.mdpi.com/article/10.3390/ph19060967/s1, Table S1: List of the primers and nucleic acid used in this study; Table S2: *m*/*z* values of peptides and their cyclic peptides; Figure S1: Purification and biotinylation of recombinant MYC protein; Figure S2: Results of gel electrophoresis after each round of selection; Figure S3: The megTYR-mediated macrocyclization was analyzed by MALDI-TOF MS; Figure S4: Binding affinity of macrocyclic peptides to bovine serum albumin (BSA); Figure S5: TAT-CP5 does not disrupt MYC–MAX interaction.
