# Supplementary material for: Discovery of Macrocyclic Peptide Inhibitors Targeting MYC Oncoprotein via mRNA Display
Source: Pharmaceuticals (Basel). 2026 Jun 22;19(6):967. doi: 10.3390/ph19060967 (PMC13304548; doi:10.3390/ph19060967)
Supplement: Supplementary file 1 [file pharmaceuticals-19-00967-s001.zip › pharmaceuticals-4242148-supplementary.pdf]

Supplementary Materials

Nomenclature used this study:

N = A, C, G, T

M=A, C

K=G, T

p = 5'-phosphorylation

Spacer 18 = Hexaethylene glycol

FAM-dT = Fluorescein-dT

Puro, Puromycin

Table S1. List of the primers and nucleic acid used in this study

Oligonucleotide name	Sequence (5’-3’)
Lib_ ssDNA-F	GTAATACGACTCACTATAGGGAGACCACAACGGTTTCCCTCTAGAAATAATTTTGTTTAACTTTAAGAAGGAGATATACCAATGTAT
Lib_ ssDNA-R	TTTTTCACCTGATCCGCTGCTAGCTGCCGCTGCCGCTGCCGCA(MNN)$_{10}$ATACATTGGTATATCTCCTTC
P-Linker	pCCCTTCACCTGATCCGCTGAAAAAAAAAAAAAAAAAA (Spacer 18) (Spacer 18) (FAM-dT) (Spacer 18) CC (Puro)
RT-PCR-F	GTAATACGACTCACTATAGGGAGACCACAACGGTTTC
RT-PCR-R	TTTTTCACCTGATCCGCTGCT

Table S2. m/z values of peptides and their cyclic peptides

Name	Synthesized AA sequence	[M+H] m/z	Name	Cyclized AA sequences	[M+H] m/z
P1	M Y G T N V V S F A C	1191.36	CP1	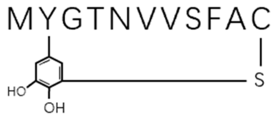	1205.36
P2	MYSEGNKCAWFSC	1525.69	CP2	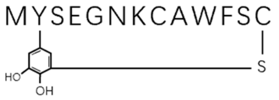	1539.69
P3	M Y Y A I K A F R V I C	1477.8	CP3	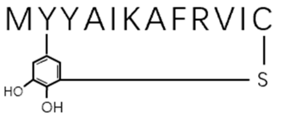	1491.8
P4	M Y R C A N A C I R F C	1450.74	CP4	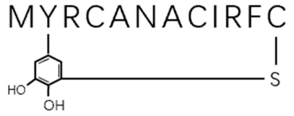	1464.74
P5	MYHVTAQWSGTDC	1498.62	CP5	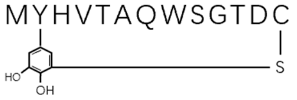	1512.62
P6	MYHLVKAFRSSSC	1528.74	CP6	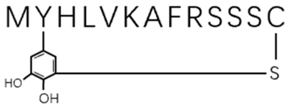	1542.74
P7	MYKPLLSTTSNRC	1513.75	CP7	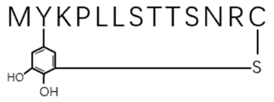	1527.75
TAT-P2	MYSEGNKCAWFSC G R K K R R Q R R R	2904.29	TAT-CP2	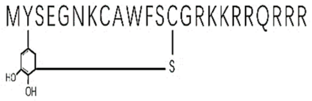	2918.29
TAT-P5	MYHVTAQWSGTDC G R K K R R Q R R R	2877.22	TAT-CP5	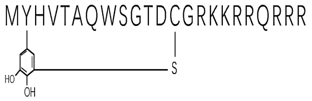	2891.22

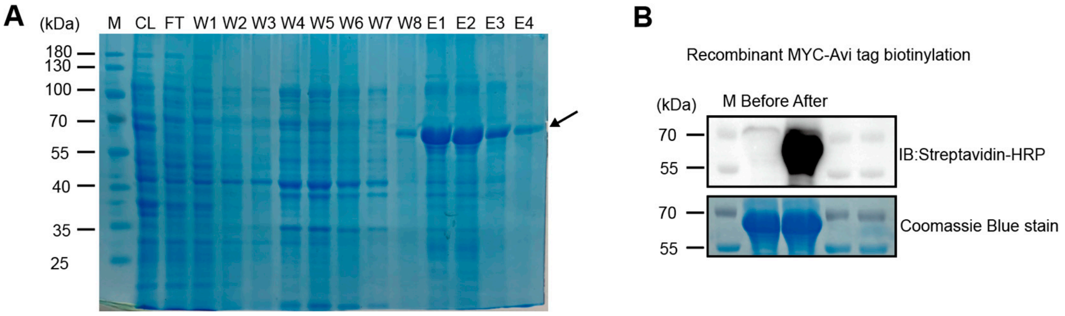

Figure S1. Purification and biotinylation of recombinant MYC protein

(A) Coomassie Blue staining of SDS-PAGE. M: Marker; CL: Cell Lysate supernatant; FT: Flow Through; W1-W3: Wash of lysate; W4-W6: Wash of wash solution (20 mM imidazole); W7-W8: Wash of wash solution (50 mM imidazole); E1-E4: elution solution (250 mM imidazole). (B) Coomassie Blue staining of SDS-PAGE and Western Blot of recombinant MYC-biotin. (Upper) Western blot of anti-Streptavidin-HRP antibody; (Lower) SDS-PAGE stained with Coomassie blue.

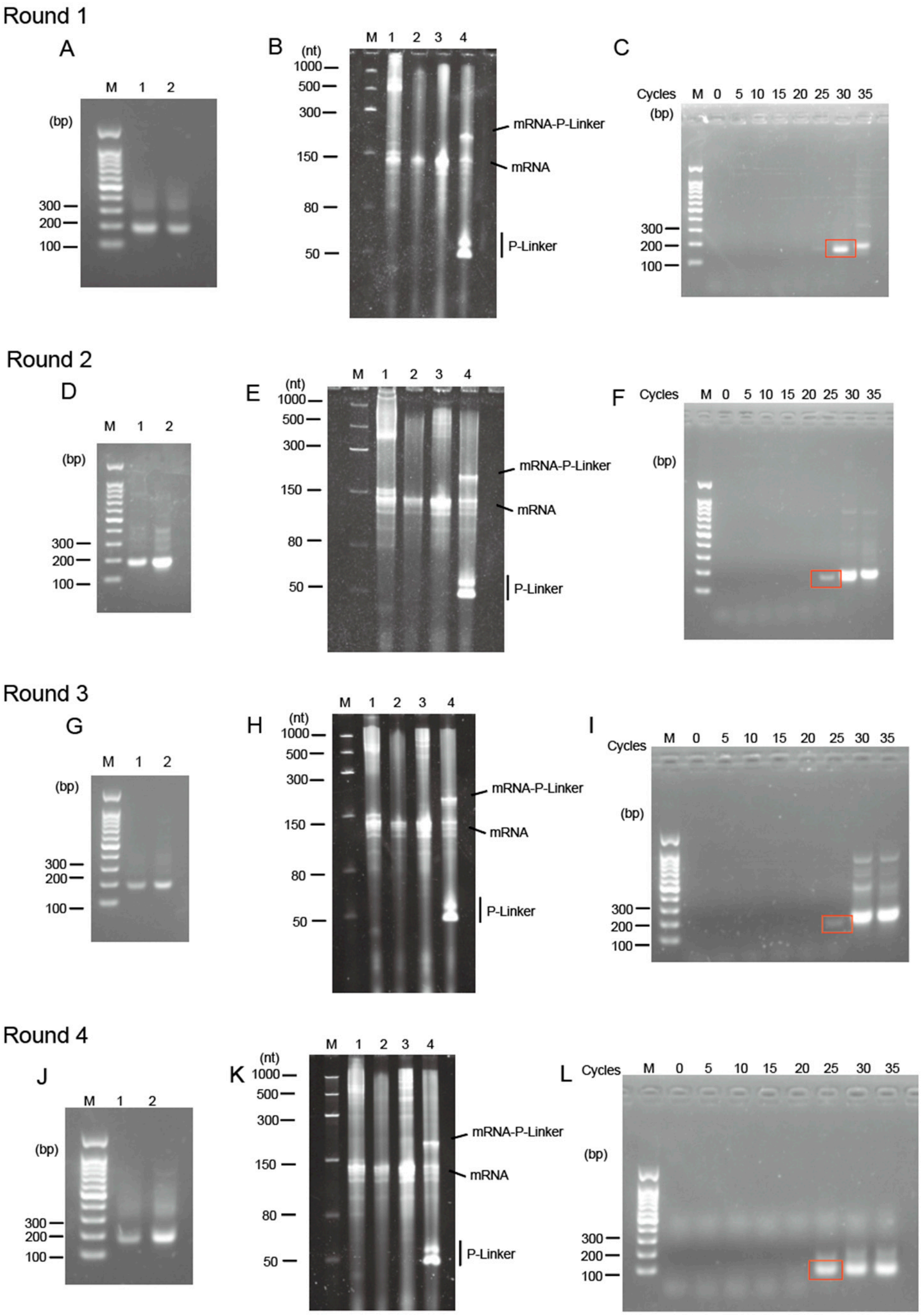

Figure S2. Results of gel electrophoresis after each round of selection

The gel images for rounds 2–4 (D–L) are shown in the same order as round 1. Round 1 is showed here as a representative example with detailed annotations: (A) 2% agarose gel electrophoresis. M: Marker; 1: PCR amplification product; 2: column-purified DNA; (B) In vitro transcription. M: Marker; 1: product of synthesized mRNA step; 2: product of DNA removal step; 3: column-purified mRNA; 4: P-Linker ligation reaction; (C): Pre-experiment for the next round of PCR. Round 1 (30 cycles); Rounds 2–4 (25 cycles).

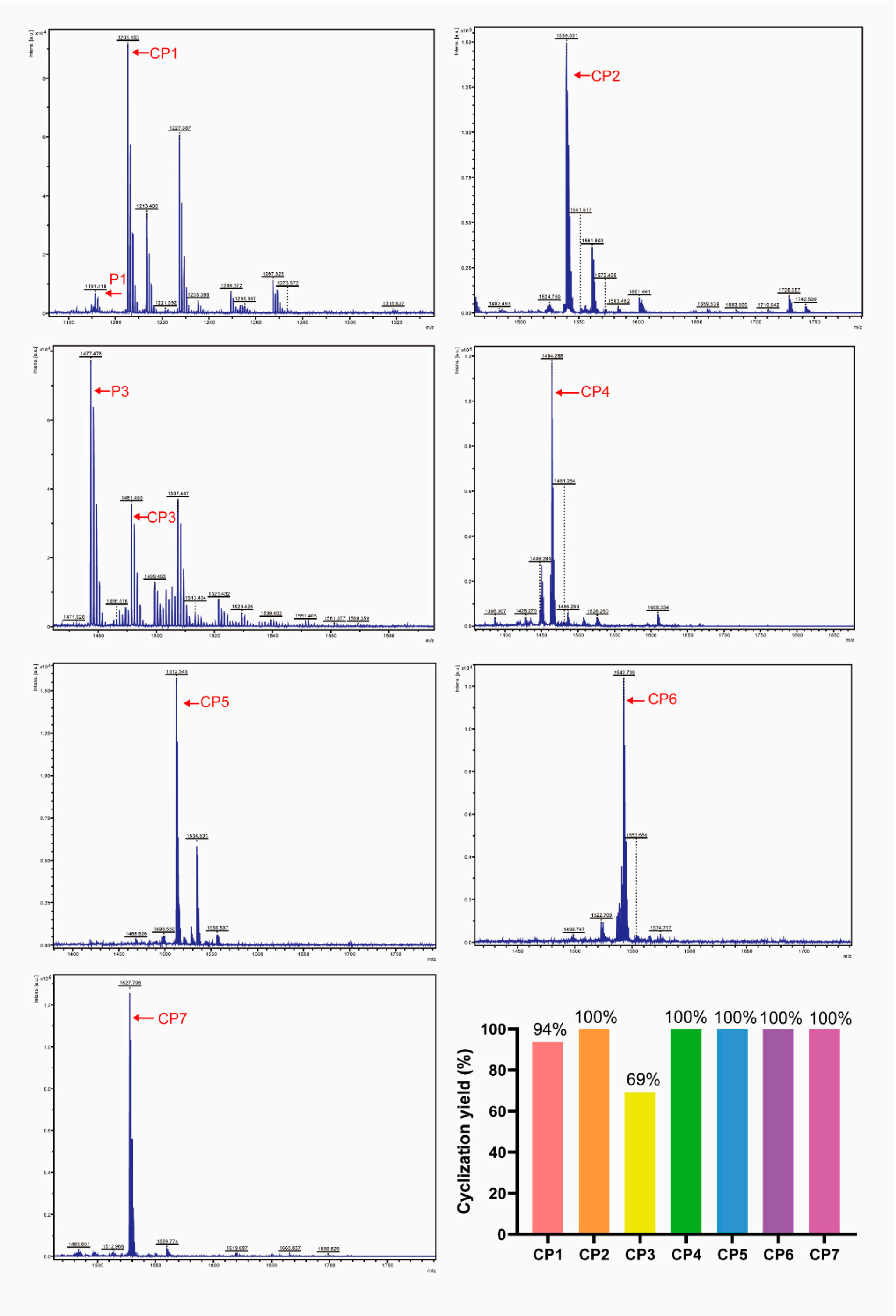

Figure S3. The megTYR-mediated macrocyclization was analyzed by MALDI-TOF MS.

The additional peaks observed in the MALDI-TOF spectra are sodium adducts ($[M+Na]^+$ and $[M+2Na–H]^+$). Their mass shifts correspond exactly to the addition of one or more sodium atoms (e.g., +22 Da for a single Na^+). And please note that CP3 was excluded from subsequent studies due to its poor cyclization efficiency.

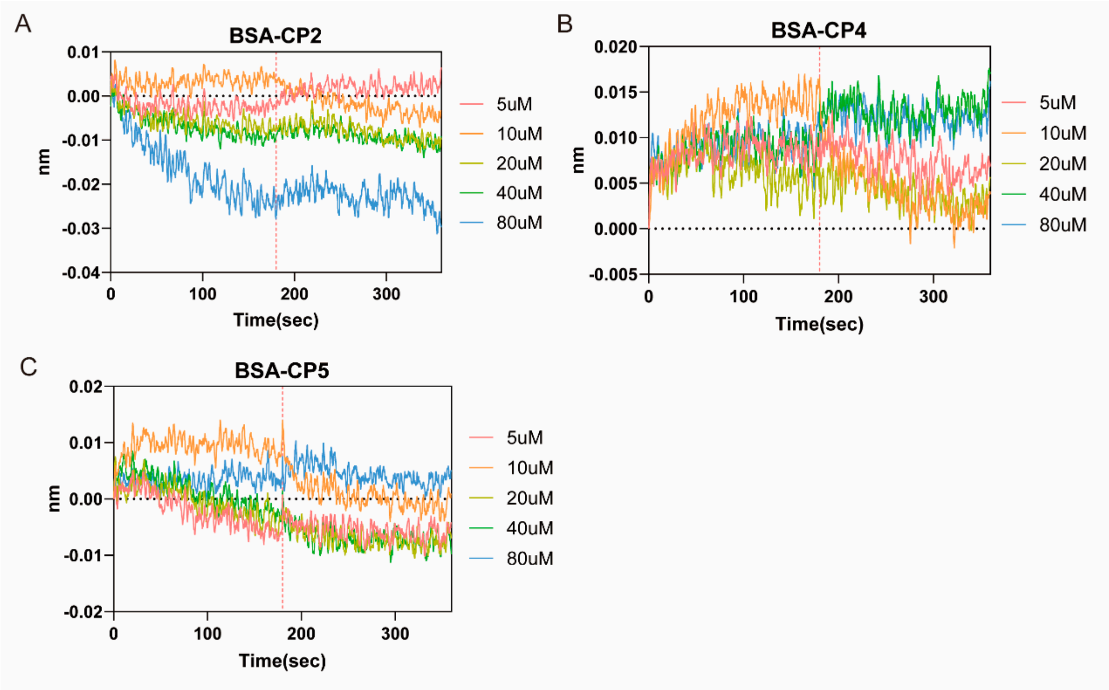

Figure S4. Binding affinity of macrocyclic peptides to bovine serum albumin (BSA)

The binding of CP2, CP4 and CP5 to immobilized bovine serum albumin (BSA) as an irrelevant control protein was measured with Bio-Layer Interferometry (BLI).

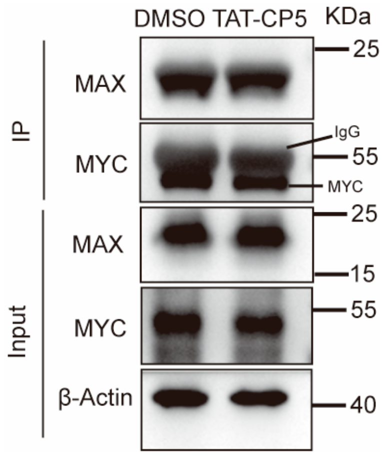

Figure S5. TAT-CP5 does not disrupt MYC–MAX interaction

HEK293T cells overexpressing Flag-tagged MYC and HA-tagged were treated with DMSO or TAT-CP5 (5 μM, 24 h). Cell lysates were immunoprecipitated with anti-HA antibody and then immunoblotted with anti-Flag antibody (for MYC protein) and anti-HA antibody (for MAX protein).
